# *In Vivo* Chromatin Organization of Mouse Rod Photoreceptors Correlates with Histone Modifications

**DOI:** 10.1371/journal.pone.0011039

**Published:** 2010-06-09

**Authors:** Caroline Kizilyaprak, Danièle Spehner, Didier Devys, Patrick Schultz

**Affiliations:** 1 IGBMC (Institut de Génétique et de Biologie Moléculaire et Cellulaire), Illkirch, France; 2 Inserm, U964, Illkirch, France; 3 CNRS, UMR7104, Illkirch, France; 4 Université de Strasbourg, Strasbourg, France; Istituto Dermopatico dell'Immacolata, Italy

## Abstract

**Background:**

The folding of genetic information into chromatin plays important regulatory roles in many nuclear processes and particularly in gene transcription. Post translational histone modifications are associated with specific chromatin condensation states and with distinct transcriptional activities. The peculiar chromatin organization of rod photoreceptor nuclei, with a large central domain of condensed chromatin surrounded by a thin border of extended chromatin was used as a model to correlate *in vivo* chromatin structure, histone modifications and transcriptional activity.

**Methodology:**

We investigated the functional relationships between chromatin compaction, distribution of histone modifications and location of RNA polymerase II in intact murine rod photoreceptors using cryo-preparation methods, electron tomography and immunogold labeling. Our results show that the characteristic central heterochromatin of rod nuclei is organized into concentric domains characterized by a progressive loosening of the chromatin architecture from inside towards outside and by specific combinations of silencing histone marks. The peripheral heterochromatin is formed by closely packed 30nm fibers as revealed by a characteristic optical diffraction signal. Unexpectedly, the still highly condensed most external heterochromatin domain contains acetylated histones, which are usually associated with active transcription and decondensed chromatin. Histone acetylation is thus not sufficient *in vivo* for complete chromatin decondensation. The euchromatin domain contains several degrees of chromatin compaction and the histone tails are hyperacetylated, enriched in H3K4 monomethylation and hypo trimethylated on H3K9, H3K27 and H4K20. The transcriptionally active RNA polymerases II molecules are confined in the euchromatin domain and are preferentially located at the vicinity of the interface with heterochromatin.

**Conclusions:**

Our results show that transcription is located in the most decondensed and highly acetylated chromatin regions, but since acetylation is found associated with compact chromatin it is not sufficient to decondense chromatin *in vivo*. We also show that a combination of histone marks defines distinct concentric heterochromatin domains.

## Introduction

Gene expression is regulated at the transcriptional level by a variety of trans-acting factors that bind to specific promoter elements to elicit transcription initiation in response to intra- and extra-cellular signals. Only a fraction of the genome is competent for factor binding and the way DNA is wrapped into chromatin regulates its accessibility thus participating actively in the regulation of gene expression [1]. In eukaryotes, DNA is packaged through a hierarchy of folding events. In the first level 146 bp of DNA are wrapped in a negative supercoil around an octamer of four pairs of the core histones H2A, H2B, H3 and H4 to form the core nucleosome whose structure is defined at the atomic level [2]. The fundamental repeated element of chromatin, the nucleosome, is composed of the core particle, linker DNA whose species-dependant average length varies between 11 to 94 bp and the linker histone H1. In low ionic strength purified chromatin appears as an extended 11 nm fiber formed by a linear beads-on-a-string nucleosomal array that compacts into 30 nm fibers in physiological ionic strength and in the presence of histone H1 [3], [4], [5]. *In vitro* reconstituted or purified 30 nm fibers are flexible and organized into imperfect helical structures [6]. Direct electron microscopy imaging of nuclear sections described highly compact electron dense heterochromatin (HC) compartments and more extended euchromatin (EC) territories but has provided little information on the organisation of chromatin beyond the nucleosomal level and in particular has not confirmed the 30 nm fibers as the fundamental secondary structure of chromatin in intact nuclei [7] [8].

This structural definition partially overlaps the biochemical and functional description of chromatin, which is separated into EC and HC on the basis of nuclease accessibility, histone modifications and transcriptional activity [9]. Transcribed genes are found associated with accessible and more readily digested EC whereas nuclease resistant HC is believed to be more compact and associated with transcriptional repressed states [10], [11], [12]. The structural origin of this increased accessibility is not fully understood and was recently challenged by the finding that coding sequences in general are more nuclease sensitive regardless of their transcriptional activity [13]. Moreover, specific post translational modifications of the core histones are associated with characteristic transcriptional states of the genome a finding which has led to the histone code concept [14]. Lysine acetylation almost always correlates with active transcription and is believed to act by neutralizing the repulsive charge interaction between DNA and the histone tails [15] and by recruiting specific chromatin associated proteins such as the nucleosome remodeling complex SWI/SNF [16], histone acetyl transferases [17] or the general transcription factor TFIID [18]. Lysine methylation is associated with distinct transcriptional states depending on which residue is modified [19]. Methylation of histone H3 lysine 4 (H3K4) or lysine 36 (H3K36) is related to transcribed chromatin whereas H3K9, H3K27 and H4K20 trimethylation generally correlate with transcriptional repression. Methylated H3K9 and H3K27 are bound by HP1 and Polycomb, respectively, which mediate chromatin compaction [20].

Sedimentation studies on recombinant nucleosomal arrays were performed to explore the link between chromatin condensation and histone modifications and showed that acetylation of H4K16 inhibits the formation of 30 nm fibers [21]. This observation is consistent with the concept that acetylation of the N-terminal tails of core histones may affect inter-nucleosomal interactions. However direct electron microscopic inspection of reconstituted chromatin fragments reveals that core histone acetylation is not sufficient to generate an open chromatin structure and that histone H1 plays a key role in this process [22]. A correlation between chromatin compaction and histone tail modifications has not been demonstrated *in vivo* at the ultrastructural level.

The aim of this study was to correlate the packing of chromatin, the transcriptional activity and the distribution of histone tail modifications in sections of cell nuclei. Here murine rod photoreceptors were investigated by electron tomography and immunolabelling to study these correlations. Mouse rod cells have an extremely dense HC domain located at the centre of the nucleus and a small EC territory placed at its periphery, close to the nuclear envelope [23]. These highly differentiated cells have packaged most of their DNA into HC but still express all house-keeping genes and undergo robust transcription of specific genes involved in the visual signal transduction pathway [24] [25]. Our findings show that nucleosomes are hyperacetylated, show higher levels of monomethylation on H3K4 and are hypomethylated on H3K9, H3K27 and H4K20 in euchromatin, and that fully extended and partially condensed chromatin fibres are associated with these modifications. The RNA polymerase II molecules are detected only in the EC territory and frequently at a fixed distance from the EC/HC interface which plays a role in the organization of transcription units. The HC compartment contains tightly packed chromatin which is organized into 30nm fibers at the periphery and into an amorphous phase in the central part. Heterochromatin nucleosomes are generally hypoacetylated and hypermethylated on H3K9, H3K27 and H4K20 however a densely packed HC territory was found hyperacetylated at the EC/HC interface thus challenging the view that acetylation is associated with an open chromatin structure. Moreover the distribution of the H4K20 and H3K9 trimethylation marks in the HC domain were different than the H3K27 mark thus defining a core and a peripheral HC compartment.

## Results

### 1-Chromatin territories in photoreceptor nuclei

**Figure 1:**
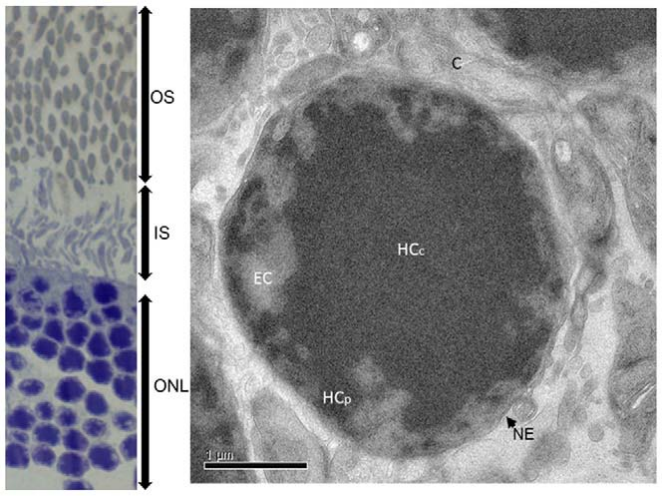
Ultrastructure of the rod photoreceptor nuclei. (A) General organization of the photoreceptor within the retina viewed by light microscopy after toluidin blue staining. ONL: Outer nuclear layer, IS: inner segments, OS: outer segments. (B) Overall view of high pressure frozen, cryo substituted rod photoreceptor nucleus observed by electron microscopy. C: Cytoplasm, EC: euchromatin, HCc: central Heterochromatin, HCp: nuclear envelope associated heterochromatin, NM nuclear envelope.

Retinas of freshly sacrificed mice were dissected according to an optimized protocol that preserves the electrophysiological activity of the tissue and were instantly cryo-immobilized by high pressure freezing to prevent chemical fixation and changes in physicochemical conditions that could reorganize the cellular ultrastructure. The retinas were then freeze-substituted, epon embedded and sectioned at room temperature. Toluïdin blue stained sections displayed the multilayered structure of the retina in which the nucleus, inner segment and outer segment of the photoreceptor were recognized (**Figure 1A**). After post staining with uranyl acetate and lead citrate, 100 nm thick retina sections observed by transmission electron microscopy revealed the characteristic appearance of rod nuclei which show a large electron dense HC domain located in their center (**Figure 1B**). Most nuclei had a nearly round shape with a maximal radius of 1.9+/−0.2 µm giving a volume of 29+/−7 µm^3^. Chromatin appeared arranged in concentric layers; the center of the nucleus being composed exclusively of HC whereas a 500–600 nm wide rim adjacent to the nuclear membrane contained a mixture of HC and less dense EC. A fraction of HC was found in close contact with the nuclear membrane thus forming a thin rim that was connected through HC bridges to the central HC. In contrast to other cell types, the EC compartment was markedly reduced in size and was confined to the nuclear periphery close to the nuclear membrane. Stereological measurements performed on 20 different nuclei showed that the HC compartment represents 71% of the surface when the nucleus is sectioned through its centre which, assuming spherical symmetry corresponds to about 17 µm^3^. The thin layer of HC in close contact to the nuclear membrane (within 150 nm from the nuclear membrane) was estimated to 3 µm^3^ or 15% of the entire HC compartment. The EC compartment contacts the nuclear membrane and projects into the central HC through multiple invaginations thus forming cavities with an extended HC/EC interface. The staining of the EC compartment showed substantial variations indicating that variable degrees of chromatin condensation coexist in this area.

### 2-Nucleosome distribution in chromatin territories

**Figure 2:**
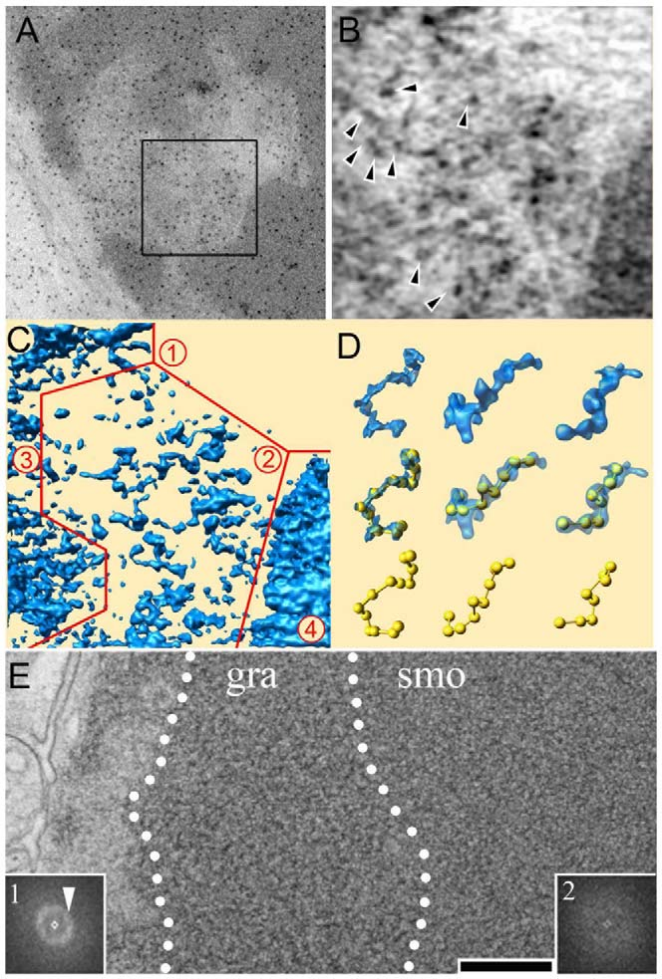
Electron tomography of rod photoreceptor nuclei. (A) Overall view of the nuclear domain in which the electron tomogram was recorded (boxed area). The dark gold beads are fiducial markers used for aligning the tilted views on a common origin (B) Four nm thick section through the tomogram showing dark electron dense particles (arrows). (C) Surface representation of the tomogram in which 4 distinct chromatin domains were delineated. (D) Extraction of characteristic 11 nm fibers from different tomograms and beads on a string representation. (E) Overall view of the heterochromatin domain showing a distinct transition between a granular (gra) and a smooth (smo) compartment. These compartments are characterized by their optical diffraction pattern shown in insert 1 and 2 for the granular and smooth territory respectively. Bar represents 300nm in (A), 100 nm in (B–C), 54 nm in (D) and 230 nm in (E).

To gain a better insight into the 3-D organisation of chromatin a 370 nm wide square area of a representative EC cavity was recorded under different viewing directions and its volume was reconstructed by electron tomography (**Figure 2A–B**). Digital sections through the reconstructed tomogram clearly revealed isolated electron dense particles whose size corresponds to isolated nucleosomes (black particles marked by arrows in **Figure 2B**). According to the abundance of these particles, the tomogram was divided into 3 distinct EC domains (areas 1–3 in **Figure 2C**) and a dense HC compartment (area 4 in **Figure 2C**). The least stained area 1 does not enclose any dense particles and probably corresponds to a nucleosome-free region at the centre of the cavity. The intensity of this area was used to set a threshold in the tomogram (4.5 σ values over the average intensity) suitable to detect of the individual electron dense particles whose number was estimated by fitting 12 nm wide spheres.

Euchromatin area 2 contains well separated particles most of which are 10 to 12 nm in size. With an average density of 58 10^3^ particles/µm^3^ they occupy about 6.7% of the volume. The particles are often connected by 2–4 nm wide threads and form extended fibers with an average spacing between particles of 22 nm (n = 49, σ = 5) (**Figure 2D**). This beads-on-a-string appearance is consistent with the structure of an extended chromatin fibre formed by core nucleosomes connected by a linker DNA filament. The estimated repeated distance of 179+/−15 bp (146 bp wrapped around the core nucleosomes and 33 bp or 11nm of linker DNA) is comparable to the 190 bp nucleosomal repeat length found in mouse spleen cells [26]. Altogether these observations show that individual nucleosomes and even connecting DNA can be resolved in the tomograms of stained nuclear sections and that individual, highly extended, chromatin fibers can be depicted in these EC domains.

Area 3 corresponds to an EC compartment which contains more densely packed particles and a nucleosome density of 145 10^3^ nucleosomes/µm^3^ was determined. Nucleosome–sized particles are distinguished at the periphery of this domain but individual chromatin fibres cannot be traced. The nucleosomes occupy about 16% of this nuclear domain which is penetrated by solvent accessible channels.

Finally, area 4 corresponds to a characteristic HC domain where the boundaries of individual particles cannot be distinguished. A total of 61% of the volume has an intensity above the threshold assigned for the nucleosomes which, assuming a similar staining than for the EC territories, yields a concentration of about 540 10^3^ nucleosomes per µm^3^. Considering the volumes of each chromatin compartment and their estimated nucleosomal densities, a total of 12 10^6^ nucleosomes are predicted to be present in the nucleus. Such an amount of chromatin would package roughly 2.3 Gbp of DNA which is only slightly lower than the sequenced mouse genome (2.5 Gbp) thus validating the estimated chromatin compaction.

Despite its electron dense nature the HC compartment showed at least two distinct textures indicative of different nucleosomal arrangements. The periphery of the HC territory had generally a granular appearance whereas the most central domain showed a highly homogeneous and smooth texture (**Figure 2E**). In order to characterize the average size of this granularity the Fourier transform was calculated for the central and external parts of the HC compartment (Inserts 1 and 2 in **figure 2E**, respectively). The central HC showed a feature-less spectrum consistent with its homogeneous texture. In sharp contrast, the peripheral granular HC region is characterized by a strong ring in the Fourier transform at 1/30.9 nm^−1^. This observation indicates that the periphery of the HC territory is formed by closely packed 30 nm chromatin fibres whereas such fibres are not detected in the most central domain.

### 3-Optimized immunolabelling of histones in condensed chromatin

**Figure 3:**
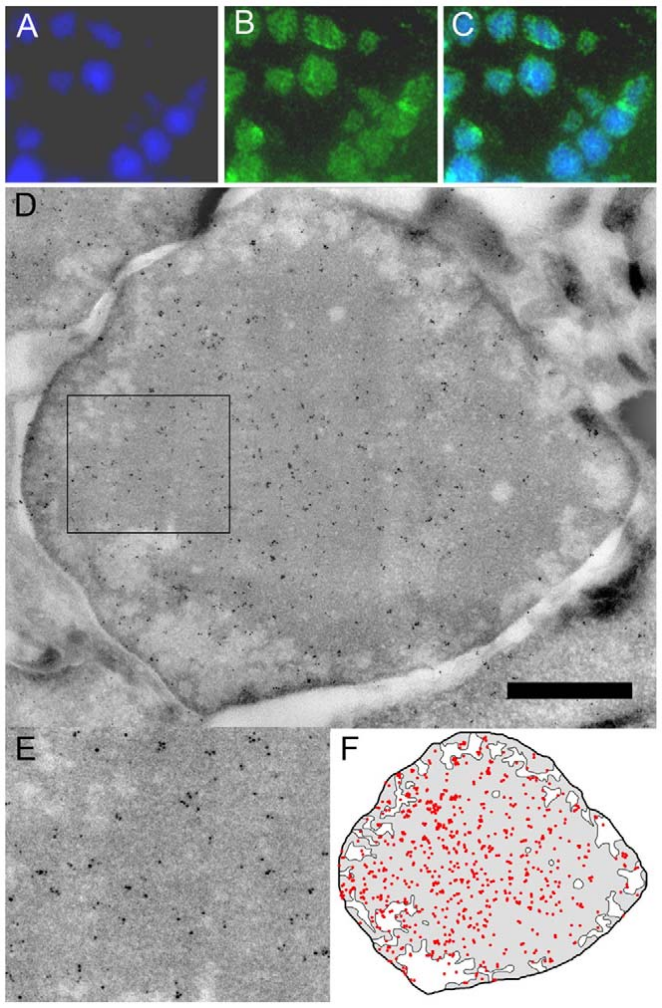
Immuno labelling of core histone H3. (A) DAPI staining of an ultra-thin cryo-section (B) Core histone H3 labelling detected by Alexa Fluor 488. (C) Merged image. (D) Overall view of the ultra-thin immunogold labelled section. (E) Enlarged region showing the uniform labelling of the section. (F) Schematic representation of the H3-specific labelling. Bar represents 1 µm in D and 0.5 µm in E.

In order to correlate the degree of chromatin compaction with the distribution of post translational histone marks we performed immunocytochemistry experiments using antibodies directed against native or modified histones. In our hands, methods using cell permeabilization failed to label the condensed central HC probably because of reduced accessibility (data not shown). Ultra-thin cryo sections of the retina were therefore produced to gain equal access to all regions of the nucleus and to optimally preserve the antigenicity of the epitopes [27]. An antibody directed against the C-terminal part of histone H3 was first used to label all nucleosomes. After immunofluorescent detection, this antibody was found to bind strongly to the central part of the cell nucleus where it overlapped with the DAPI staining thus indicating that the condensed HC domains are unveiled by the sectioning process and accessible for antibody binding (**Figure 3A–C**). In order to quantify the labelling density and its variations in the different nuclear domains, the primary antibody was immuno-gold labelled and the sections were observed by electron microscopy to determine the number of electron dense particles per surface area (**Figure 3D–F**). For the anti H3 antibody the labelling density was of 86 particles per µm^2^ (p/µm^2^) in HC and of 42 p/µm^2^ in EC yielding a HC/EC labelling ratio of 2. This ratio is significantly lower than the HC/EC nucleosome density ratio of at least 5 found by tomography suggesting that the accessibility of this epitope is affected by the nucleosomal packing. To quantify the radial variation of the labelling density within the nucleus, the HC domain was contoured and the gold particles were counted in concentric rings of decreasing size, each separated by 150 nm (**Figure 8**). The labelling density is remarkably uniform for the anti H3 antibody throughout the whole HC.

### 4-Different trimethylation states of silenced chromatin

The distribution of three histone trimethylation marks specific for transcriptionally silenced chromatin were investigated by using antibodies recognizing histone H3 modified on lys 9 (H3K9me3), on lys 27 (H3K27me3) or histone H4 modified on lys 20 (H4K20me3).

**Figure 4:**
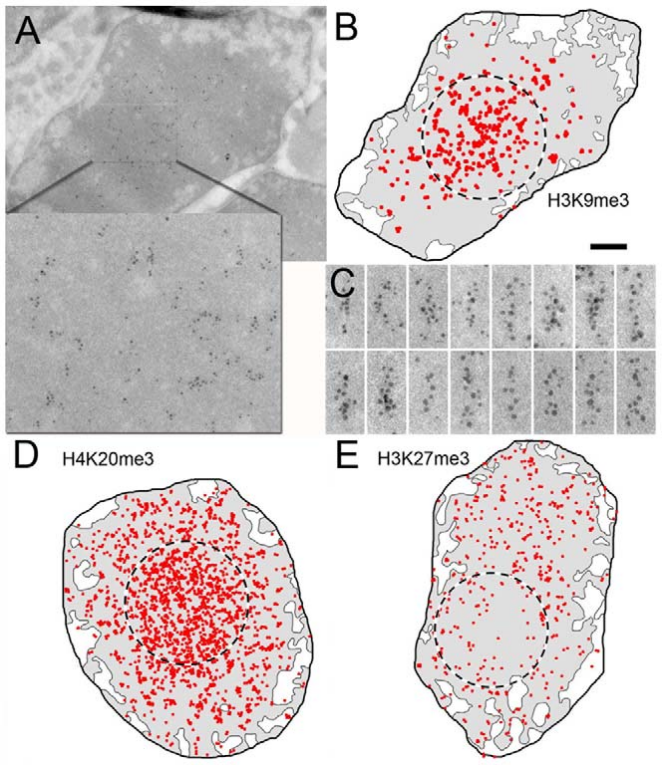
Immunolabelling of transcriptionally inactive histone marks. (A) Overall view of a rod nucleus immunogold labelled with H3K9me3-specific antibodies (B) Schematic representation of the H3K9me3-specific labelling. The dashed circle indicates the more intensely labelled central HC core (C) Enlarged region showing the distribution of the labelling along 30 nm fibers. (D) Schematic representation of the H4K20me3-specific labelling. The dashed circle indicates the more intensely labelled central HC core (E) Schematic representation of the H3K27me3-specific labelling. The dashed circle indicates the less labelled central HC core. The bar represents 500 nm in (A, B, D, and E) and 85 nm in (C).

The H3K9me3 mark is strongly underrepresented in the EC regions since the labelling density is 15 times lower than the average HC labelling. In addition, this mark is not homogeneously distributed over the HC domain since the labelling density increases towards the center of the HC (**Figure 4A and B**). The gold particle density was 40 p/µm^2^ in the most external HC ring (from 0 to 150 nm from the EC/HC interface) and increased regularly to reach 255 p/µm^2^ in the center of the nucleus (**Figure 8**). This 6.4 times increased labelling of the inner HC is highly significant since it contrasts with the control H3 labelling which varied by less than 5%. Direct inspection of the labelled nuclei identifies two distinct HC domains. In a round almost central core HC domain representing about 25% of the total HC surface, the labelling density is 245 p/µm^2^ whereas in the peripheral HC it falls to 42 p/µm^2^. Interestingly, both in the peripheral and in the core HC, the gold labels were often arranged along 30 nm wide fibers indicating that some chromatin fibers can bind several antibodies whereas neighboring fibers cannot (**Figure 4C**). The absence of labelling is not due to less accessible epitopes or to an unfavorable cutting plane since such a pattern was not observed for the control anti H3 antibodies. This fiber pattern discloses a spreading mechanism where the nucleosomes of some fibers are highly trimethylated on H3K9.

The overall distribution of the H4K20me3 mark is comparable to that of the H3K9me3 modification. The HC domain is on average 11.6 times more labelled than the EC compartment and a 5.6 fold increase in labelling density is observed between the most peripheral and the most central parts of the nucleus. A core HC domain, which represents 42% of the nuclear radius, can be defined in which the labelling is almost homogeneous (400 p/µm^2^) and 2.8 times higher than in the periphery (140 p/µm^2^) (**Figure 4D** **and** **8**). A fiber-like labelling pattern is also observed for the H3K20me3 mark.

The H3K27me3 marks are also enriched in the HC region but in contrast to the previously analyzed trimethylations, show a decreased labelling from the periphery to the center of the HC territory (**Figure 4E** **and** **8**). A central HC core domain representing about 25% of the HC surface can thus be delineated in which the average labelling density is 24 p/µm^2^ whereas it rises to 87 p/µm^2^ in the periphery.

### 5-Condensed chromatin can be acetylated

**Figure 5:**
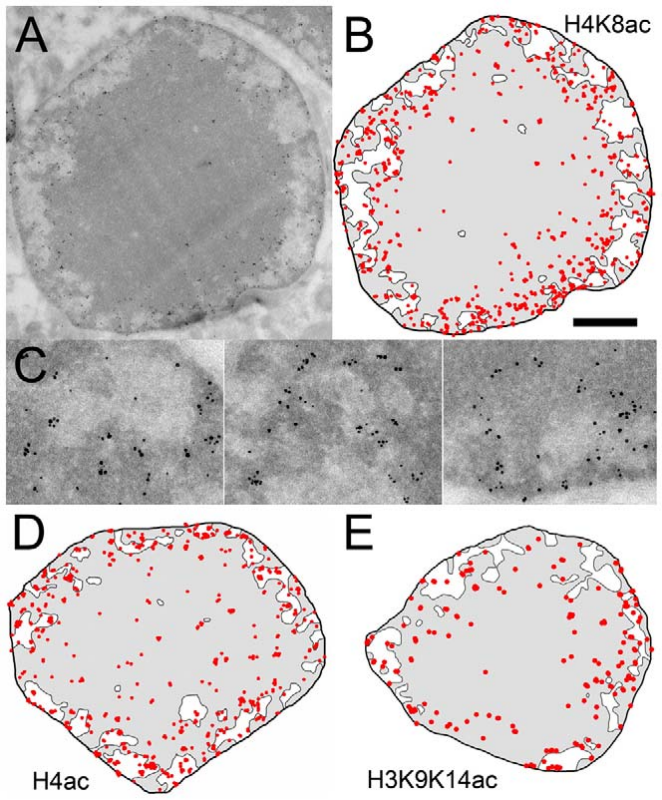
Immunolabelling of acetylated histone marks. (A) Overall view of a rod nucleus immunogold labelled with acetylated H4K8-specific antibodies. (B) Schematic representation of the H4K8ac-specific labelling. (C) Enlarged region showing the distribution of the H4K8ac labelling around the EC cavities. (D) Schematic representation of the labelling for the pan-actetylated H4. (E) Schematic representation of the labelling for H3 actetylated on K9 and K14. The bar represents 1 µm in A, B, D, E, and 260 nm in C.

The distribution of acetylated histones generally associated with actively transcribed genes and with decondensed chromatin was investigated using an antibody directed against acetylated H4K8. In sharp contrast to the silencing marks, H4K8ac is almost absent in the central HC domain (4 p/µm^2^) but, unexpectedly, is found highly enriched in the condensed HC closest to the HC/EC interface (97 p/µm^2^) and in the EC compartment (41 p/µm^2^) (**Figure 5A–C** **and** **8**). The nucleosomes present in the EC and close to the EC/HC interface are thus hyperacetylated on histone H4. The condensed chromatin associated with the nuclear membrane is also heavily decorated by this antibody (100 p/µm^2^). An antibody directed against all acetylated forms of H4 gave the same labelling profile (**Figure 5D**).

The distribution of acetylated histone H3 was investigated by using an antibody specific for acetylated K9 and K14. Despite a slightly lower labelling density, the distribution of H3K9K14ac is similar to that of H4K8ac (**Figure 5E**). Highest labelling is found at the EC/HC interface and in EC domains where less condensed chromatin is more likely to occur (**Figure 8**). These observations confirm that the extended chromatin is highly acetylated and show that the highly condensed chromatin placed at the interface with EC is also acetylated thus demonstrating that this histone mark is not sufficient to promote chromatin decondensation.

### 6-Euchromatin and its interface with heterochromatin show high levels of H3K4 monomethylation

**Figure 6:**
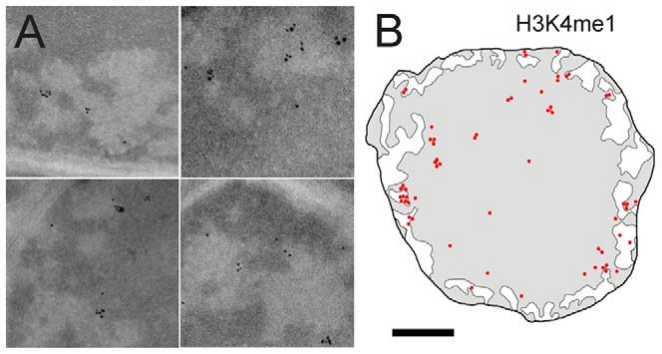
Immunolabelling of lysine 4 monomethylated histone H3. (A) Enlarged region showing the distribution of the H3K4me1 labelling around and within the EC cavities. (B) Schematic representation of the labelling for the H3K4me1 mark. The bar represents 260 nm in A and 1 µm in B.

Unlike the H3K9, H3K27 or H4K20 methylations described above, the methylation of lysine 4 of histone H3 is a post translational modification exclusively associated with actively transcribed genes and particularly with the early transcribed region [28]. The distribution of this mark was investigated using an antibody directed against the monomethylated form of H3K4. Despite a lower labeling density than observed for the previous marks, H3K4 monomethylation is 4 time more abundant in EC than in the most central part of the HC domain and is found enriched in the condensed HC closest to the HC/EC (**Figure 6** **and** **8**). The distribution of the methylation mark is thus comparable to that of the acetylation mark with the notable difference that the enrichment in the EC compartment is more important for H3K4me. Due to the lower nucleosomal content in the EC compartment acetylation density drops by a factor of 2.1 between the HC close to the HC/EC interface and the EC. In the case of H3K4 methylation, the labeling density remains constant suggesting that the extended chromatin is more enriched in H3K4 monomethylation than in acetylation marks.

### 7-RNA polymerase II accumulates close to the EC/HC transition

**Figure 7:**
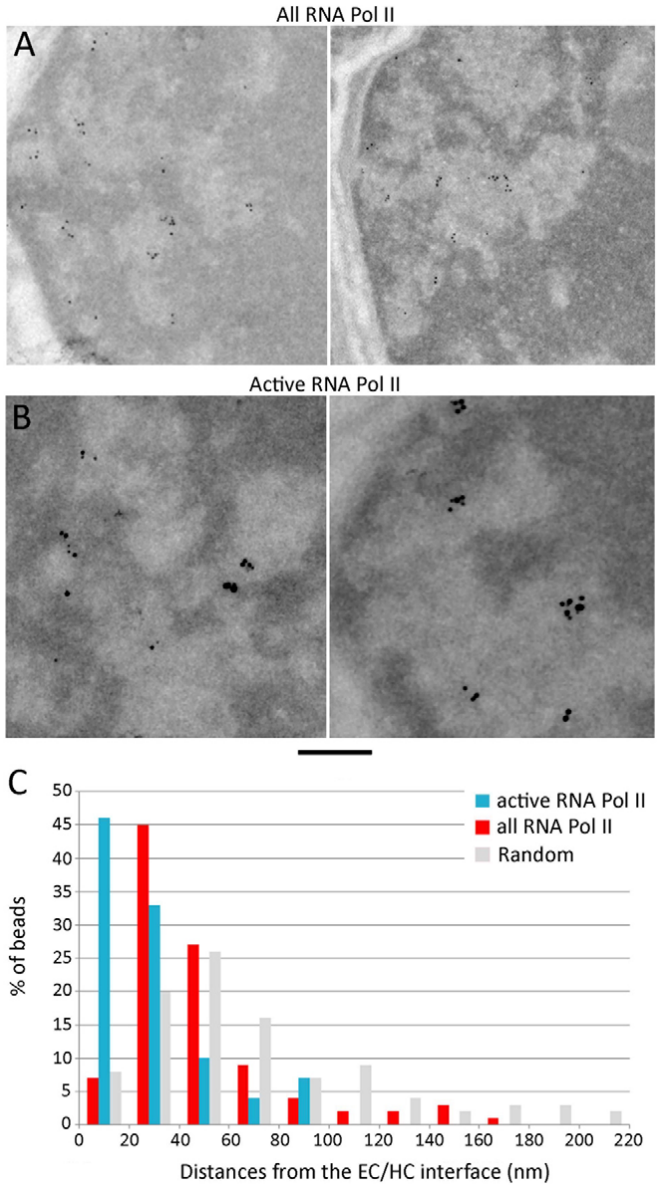
Immunolabelling of RNA Polymerase II. (A) Rod nuclei immunogold labelled with antibodies directed against the total pool of RNA Pol II molecules. (B) Rod nuclei immunogold labeled with an antibody directed against the transcriptionally active RNA Pol II in which the CTD heptapeptide is phosphorylated on Ser5. (C) Histogram showing the distances between the EC/HC interface and the immunogold particles in the case of the total pool of RNA Pol II (blue) and the actively transcribing RNA Pol II (red). The grey histogram represents a simulated random distribution of the particles in the same EC domains. The bar represents 200 nm.

In order to position RNA polymerase II (RNA Pol II) molecules an antibody targetting the repeated heptapeptide of the C-terminal domain (CTD) of the largest RNA Pol II subunit was used. This antibody recognizes all forms of RNA Pol II since it is directed against the non phosphorylated CTD. The RNA Pol II were found almost exclusively (79%) within the EC compartment where the labelling was 14.5 times higher than in the HC domain. Interestingly the labelling was not distributed randomly within the EC cavities but appeared to be stronger close to the EC/HC interface (**Figure 7A**). To quantify this effect, the distances of the gold beads to the EC/HC interface were plotted as a histogram along with a modeled random distribution of beads to take into account the size distribution of the EC cavities (**Figure 7C**). This representation shows that the RNA Pol II distribution peaks at a distance of 40 nm from the interface and 67% of the label is found within 40+/−20 nm. In case of a random distribution, the plot is flatter and a maximum is observed at 55 nm that contains only 26% of the beads. The most central parts of the cavities contain only 8% of the RNA Pol II labelling when it should be 23% if random. These experiments indicate that RNA pol II molecules accumulate in the EC domain close to the EC/HC interface and are depleted in the center of the EC cavities.

**Figure 8:**
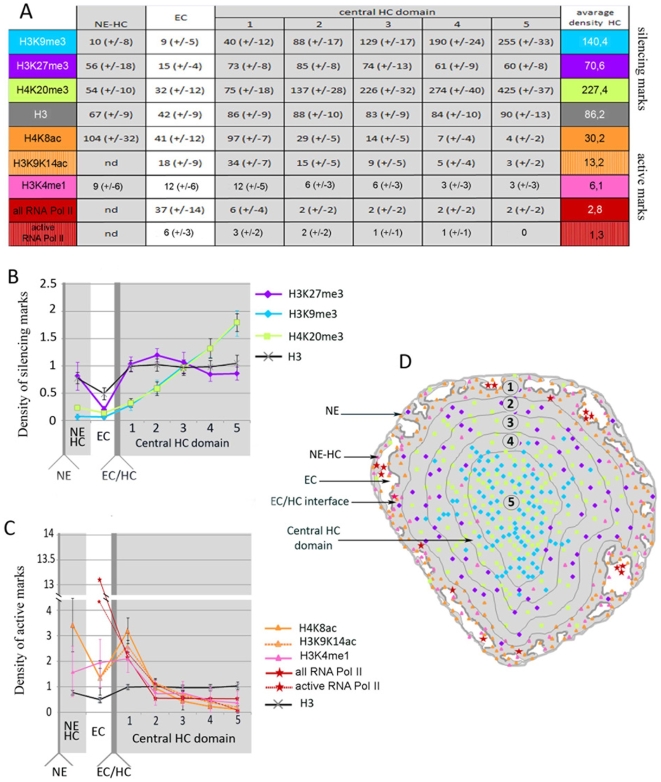
Radial distribution of the labelling densities within rod nuclei. (A) Table showing the labelling densities found for each antibody in the different chromatin compartments. (B) Graph representing the labelling density of the silencing histone marks in concentric nuclear compartments. From left to right the graph represents the nuclear envelope-associated heterochromatin (NE-HC), the euchromatin compartment (EC) and five concentric rings of the central heterochromatin domain that are separated by 150nm as indicated on panel B. The darkest bar positions the EC/HC interface. The labelling densities were normalized by the average labelling density of the central HC domain. (C) Same representation as in (B) for the histone marks specific for active transcription. (D) Schematic representation of a rod nucleus showing the euchromatin (white) and the heterochromatin (grey) territories. The central heterochromatin domain is separated into 5 concentric rings separated by 150 nm and analyzed independently to determine the radial variation in labelling density. The coloured dots represent schematically the distribution of the histone marks and respect the colour code used in panel (A).

In order to investigate the distribution of the transcriptionally active form of RNA Pol II an antibody directed against the CTD phosphorylated on serine 2 of the heptapeptide repeat. The active RNA Pol II is located preferentially (enriched 4.3 times) in the EC compartment. The histogram representing the distance of the active polymerases to the EC/HC interface shows that 66% of the active enzyme peaks at 20+/−20 nm from the interface. Moreover no active RNA Pol II labeling could be detected in the most central parts of the EC cavities. These results show that the transcribing enzyme is located even closer to the interface than the total pool of RNA Pol II.

## Discussion

Murine rod photoreceptors are terminally differentiated cells and contain a highly condensed HC domain placed at the center of the nuclei. The small size of the EC compartment, the peculiar chromatin organization and the availability of genome wide gene expression data turn these cells into unique models to study the correlations between chromatin compaction, transcriptional activity and histone modifications. At first sight, rod photoreceptor nuclei show two distinct nuclear domains that reflect the early structural definition of EC and HC based on regions differentially stained with basophilic dyes [29]. Our results show that this binary EC/HC concept is oversimplified and reveal a variety of chromatin states in both nuclear compartments. A specific pattern of histone modifications is associated to each chromatin state and transcriptional activity is confined to the decondensed chromatin areas.

### The 30nm chromatin fiber forms a discrete level of heterochromatin compaction

A widely accepted model for chromatin compaction involves successive folding events during which linear nucleosome arrays wrap into 30 nm wide helical fibers forming either a solenoid or a zig-zag assembly. Mostly observed *in vitro* for dispersed chromatin, 30 nm fibers are more elusive in the cellular context except for isolated starfish sperm or chicken erythrocyte nuclei placed in hypotonic conditions [30]. Direct evidence that such fibers are fundamental structural units of condensed chromatin in physiological ionic strength comes from early small angle X-ray scattering experiments of isolated nuclei which showed a diffraction signal at 1/30nm^−1^ proposed to be generated by tightly packed chromatin fibers [31]. Such a signal was however never detected in electron micrographs of sectioned cell nuclei thus raising the possibility that it could originate from structures other than chromatin. Technical reasons such as poor structural preservation or lack of repeated units were eluted by the observation of frozen hydrated cryo sections which preserve optimally the cell architecture. The absence of any detectable signal for 30nm fibers in frozen hydrated section of mitotic chromosomes [32] [33] or of cultured cells nuclei [8] led the authors to support a model in which the fibers are highly interdigitated thus leading to a liquid crystal-like organization of nucleosomes.

Our studies revealed for the first time a signal at 1/30nm^−1^ in the Fourier transform of nuclear images. The highly compact nature of the rod nucleus may have favoured the detection of the fibers and special care was taken to perturb as little as possible the nuclear organisation during specimen preparation. In this respect, the nuclei were not isolated from cells, the physiological ionic strength was preserved during dissection and the freshly dissected tissue was shown to be functional just prior to high pressure freezing. Interestingly 30nm fibers are only detected in the periphery of the HC whereas the central part appears uniform and does not show any periodic signal at 1/30nm^−1^. Our results thus favour a model in which rod HC is organized as closely packed 30 nm fibers which, consistent with the liquid crystal model, appear melted in the most central HC. However, even in these central parts of the nuclei, our immunolabelling data reveal 30 nm wide fibers, suggesting that the diffusion of nucleosomes around the positions they would adopt within a regular 30 nm fiber is limited. The transition between the homogeneous and the granular HC is unrelated to histone acetylation but correlates with a specific trimethylation pattern that could contribute either directly or through the recruitment of specific factors to interfiber interactions.

### Functional organization of rod heterochromatin into concentric layers

The rod HC is organized into three concentric layers, each characterized by a specific combination of histone modifications and this arrangement reflects the functional layout of the nuclei. The central HC core has a radius of 0.8 µm corresponding to 42% of the nuclear radius and contains histones highly enriched in H4K20me3 and H3K9me3 marks, weakly trimethylated on H3K27 and deacetylated. The size of this domain correlates with Fluorescence In Situ Hydridization (FISH) experiments showing that telomeric, centromeric and subcentromeric satellite DNA are found between 0 and 45% of the nuclear radius [34]. The core HC thus shows all the hallmarks of constitutive heterochromatin. Interestingly both H4K20me3 and H3K9me3 marks are distributed along 30 nm fibers indicating their accumulation on contiguous genomic locations consistent with their spreading around an initial deposition site. Self propagation is a characteristic feature of centromeric HC in mammals where spreading is based on the SUV39H methylase that favours the binding of HP1 proteins which recruit more methylases and propagate the H4K20me3 mark [35].

A peripheral domain surrounds this central core and extends to about 0.2 µm to the EC/HC interface which corresponds to 42% and roughly 60–80% of the nuclear radius since the interface is folded. In this domain the histone tails are not acetylated and show reduced H4K20me3 and H3K9me3 and higher H3K27me3 levels than the central core. The H3K27me3 mark was associated in several systems to facultative HC defined here as genomic regions that have the opportunity to adopt an open or a compact conformation within defined temporal or spatial constraints [36]. For example, H3K27me3 has an important role in embryonic development and was found to be involved in long range HOX gene silencing through the Polycomb group of proteins [37]. In rod nuclei the accumulation of H3K27me3 around the central core is consistent with a developmentally regulated onset of these marks [34].

Finally a 0.2 µm thick rim of heterochromatin at the EC/HC interface displays the same histone trimethylation pattern than the peripheral HC domain but is surprisingly highly acetylated. FISH experiments have located the gene rich regions of the genome, irrespectively of their transcriptional status, between 70 and 100% of the nuclear radius [34]. This layer of the rod nucleus is highly heterogeneous and contains intermingled Eu- and Heterochromatin but it is tempting to speculate that transcriptionally inactive genes are located in this compact but acetylated HC territory whereas actively transcribed genes are placed in the adjacent EC territory. Our results show that the role of histone acetylation needs to be reassessed since it does not primarily affect the tight nucleosomal packing and can be associated with inactive chromatin. The position of these marks at the HC/EC interphase raises the possibility that these marks contribute to the general organization of the nucleus by partitioning the gene rich and gene poor regions of the genome into distinct territories.

### Transcriptionally active chromatin

Transcription, as probed by the presence of RNA Pol II, is strictly restricted to the EC compartment, which is characterized by the presence of decondensed chromatin, acetylated and monomethylated histone tails, and the absence of H3K9me3, H3K27me3 and H4K20me3 marks. Hyperacetylation of H3 and H4 tails is a hallmark of actively transcribed genes in higher eukaryotes [38], [39], [40] and *in vitro* transcription experiments indicate that acetylation facilitates chromatin unfolding and transcription [41]. However, recent folding studies showed that acetylation of the H4 tail does not totally impair condensation and that histone H1 eviction is necessary to form an extended chromatin structure [22]. Accordingly, our *in vivo* observations confirm that H3 and H4 acetylation on its own is not sufficient to elicit chromatin decondensation at the HC/EC interface. *In vitro* the extended 11 nm chromatin fibers showing the typical beads-on-a-string appearance were only observed in the absence of the histone H1, which is believed to stabilize the 30 nm fiber by acting on the path of DNA at the exit of the nucleosome [42], [43]. Therefore our observations indicate that the removal of H1 is a critical event in the transition towards an active transcriptional state.

Electron tomograms revealed large variations in nucleosomal packing in the EC compartment ranging from extended 11 nm fibers to more densely packed chromatin regions. These variations suggest that all the euchromatin is not in the same transcriptional state and that significant transcriptional regulation may still occurs in the EC compartment through modulation of the chromatin structure. The fraction of the genome exposed in the EC territory can be roughly estimated to be 20 Mbp or less than 1% of the mouse genome from the average nucleosome density (about 10^5^/µm^3^) and the volume of the EC compartment (about 9 µm^3^). This fraction may correspond to genes that can rapidly switch between an activated and a repressed state.

The distribution of the actively transcribing RNA Pol II correlates with the EC/HC interface since both the phosphorylated and the total pool of RNA Pol II accumulate at a defined distance to the HC compartment. These distance constraints, which are found to be more stringent for the active enzymes, indicate that the transcription process takes place preferentially at 20 nm or 1–2 nucleosomes away from the interface. The significance of this observation is still unclear but it is tempting to speculate that chromatin fibers loop out of the HC domain and that transcribing polymerases are located close to the basis of these loops.

## Materials and Methods

### Retina dissection

The ICS/IGBMC animal facility is approved by the French Ministry of Agriculture and Fisheries' veterinary services (D67-218-5 - notification of 13/10/2008). The experiments involving animals were conducted under the supervision of a staff member holding an animal experimentation authorization from the Ministry of Agriculture and Fisheries: having followed the obligatory national training programs for animal handling, including procedures of euthanasia. The institution is guided by the International Guiding Principles for Biomedical Research Involving Animals developed by the Council for International Organizations of Medical Sciences and all experiments were performed in accordance with the National Institutes of Health *Guide for the care and Use of Laboratory Animals*.

Thirteen to twenty-one weeks old C57/BL6 mice were sacrificed in accordance with the National Institutes of Health *Guide for the Care and Use of Laboratory Animals*. Their eyes were rapidly enucleated and placed in Artificial CerebroSpinal Fluid (ACSF) buffer (126 mM NaCl, 2.5 mM KCl, 1.2 mM MgCl_2_, 2.4 mM CaCl_2_, 1.2 mM NaH_2_PO4, 18 mM NaHC0_3_, 11 mM Glucose, PH 7.4, equilibrated with 95% O_2_ and 5% CO_2_) for the dissection. The retina were gently separated from the pigment epithelium and the sclera after removal of the lens, iris and vitreous humor. The dissection was completed in less than 5 min [44].

### High Pressure Freezing and freeze substitution

For High Pressure Freezing (HPF), retinas were punched (Miltex, biopsy punch) to obtain an oriented disk 1.5 mm in diameter that fits into the specimen carrier. After infiltration in a drop of cryoprotectant containing 10% dextran (Sigma # D1662) and 10% BSA (Sigma # A4503) v/v in ACSF buffer (5 sec), each disk was placed onto a 200 µm thick flat gold-plated specimen carrier in order to be vitrified in the HPF machine (EMPACT2, Leica Microsystems, Vienna).

After HPF, the samples were freeze substitute [45] at −90°C for 80 h in acetone supplemented with 2% OsO_4_ and were then warmed up slowly (1°C/h) to −60°C in an Automated Freeze Substitution device (AFS2, Leica Microsystems). After 8–12 h the temperature was raised to −30°C (1°C/h) and the samples were kept at this temperature for 8–12 h before being rinsed several times in acetone. The samples were then infiltrated with gradually increasing concentration of Epon in acetone (1∶2, 1∶1, 2∶1 volume ratio and finally pure Epon) for 2–3 h while raising the temperature. Addition of pure Epon was performed at room temperature. After polymerization of the resin at 60°C, 70–100 nm thin sections were produced using an ultramicrotome (ultracut UC6, Leica Microsystems, Vienna), collected on formvar-carbon coated hexagonal 50 mesh copper grids and post-stained for 5 min with 2% aqueous uranyl acetate, rinsed and incubated for 2 min with lead citrate. The quality of the vitrification was assessed by the absence of recognizable ice crystal ghosts.

#### Stereology

To determine the proportion of the nuclear volume occupied by the different chromatin compartments we used stereological methods [46] and approximated the nucleus as concentric spheres composed, from inside to outside, of core HC, HC, EC, and membrane interacting HC. To estimate the volume of the nuclei, the radius of 20 sectioned nuclei were measured and plotted as a histogram. The peak of the histogram was close to the largest measured value and gave the radius of the central section of the nuclei. The volume occupied by a given chromatin compartment is proportional to the surface occupied by this compartment in a central section of the nucleus corrected for its radial distribution. The areas of the different chromatin compartments were determined by applying an intensity threshold on low pass filtered images. The surface of the central HC compartment was used to determine an average HC radius that was used to calculate the volume. Heterochromatin was considered to be associated to the nuclear envelope (NE) when it was within 150 nm of the NE. The volume of this shell was calculated and multiplied by the fraction of this area occupied by NE-HC to obtain the volume of NE-HC.

#### Electron Tomography

Ten nanometer colloidal gold particles were applied on one side of the grid to be used as fiducial markers to align the tilt series. The specimen was inserted in a transmission electron microscope equipped with a field emission gun and operating at 200 kV (Tecnai F20, FEI Company). Digital images were recorded at a magnification of 29.000× on a Pelletier cooled 2k CCD camera leading to a pixel spacing of 3.6 Å (Gatan, Inc., Pleasanton, CA, USA). Each tilt series was recorded in bright-field mode over a tilt range of −65° to +65° with 1° increments giving a total of 131 images automatically collected with the Xplore 3D software (FEI Company). The recorded images were aligned on a common origin by means of cross correlation techniques and the 3-D reconstruction was performed using the Inspect3D tomography reconstruction package (FEI Company) according to the weighted-back projection methods or the Simultaneous Iterative Reconstruction Technique (SIRT). Surface rendering was performed by gray level thresholding using the Chimera software (http://www.cgl.ucsf.edu/chimera) after having low pass filtered the tomograms to 1/10 nm^−1^. In order to position the electron dense particles the low pass filtered tomograms were searched for peaks of high intensity separated by more than 12 nm. For modeling, a 12 nm sphere was placed at each peak coordinate.

#### Antibodies

Active marks of transcription were detected with rabbit polyclonal antibodies directed against H3K9K14ac (Millipore # 06-599), H4K8ac (Abcam # ab15823, Cambridge), pan acetylated H4 (Millipore # 06-866) and H3K4me1 (Abcam # ab8895). Inactive marks of transcription were detected with rabbit polyclonal antibodies directed against H4K20me3 (Abcam # ab9053), H3K27me3 (Millipore # 07-449) and H3K9me3 (diagenode # pAb-056-050). Total Histone H3 was detected with a rabbit monoclonal antibody directed against the C-terminus of histone H3 (Millipore # 05-928). RNA polymerase II was detected with an IgG1-type mouse monoclonal antibody developed at IGBMC (1PB7C2). This immunopurified antibody reacts with the conserved heptapeptide repeat of the largest subunit of eukaryotic RNA polymerase II. The active form of RNA polymerase II was detected with an IgM-type mouse monoclonal antibody directed against the phosphoserine 2 in the heptapeptide repeat of Rpb1 (Covance # MMS-129R). The primary antibodies were revealed with goat anti-rabbit ultra-small gold-conjugates (Aurion, Wageningen, The Netherlands). For the detection at the light microscope level goat anti-rabbit IgG coupled to AlexaFluor 488 were used (Invitrogen # A11008).

#### Immunocytochemistry

Retina samples were prepared, sectioned and immunolabelled according to Tokuyasu [47]. *Sample preparation:* Briefly, after dissection, retina were cut into small blocks of about 1mm3, fixed in 2.5% formaldehyde, 0.1% glutaraldehyde in 0.1 M Phosphate Buffer Saline pH 7.4 (PBS) for 1h. Free aldehydes were blocked with several washes in PBS supplemented with 50 mM glycine. The blocks were infiltrated with 2.3 M sucrose in PBS in an Eppendorf tube on a rotating wheel for at least 2 h at 4°C. The blocks were then mounted on the holders of the cryoultamicrotome and rapidly frozen in liquid nitrogen. *Cryosectioning:* Ultra-thin cryo-sections (100 nm) were cut using a cryo-ultramicrotome (UM6, Leica Microsystems) at −130°C using diamond knives (Diatome, Switzerland) and recovered with a drop of 2.3 M sucrose on formvar-carbon coated 50 mesh nickel grids or on coated light microscope slides for immunofluorescence. After thawing, immunolabelling was performed using the automatic Immuno Gold Labelling apparatus (IGL, Leica Micosystems, Vienna). *Immunolabelling:* After a blocking step of two times 10 mn in glycine (0,5 mM in PBS) and another 10 mn in 1% BSA/PBS (blocking buffer), the sections were incubated 20 min with the primary antibodiy. After 4 times 2 min washes on 10× diluted blocking buffer bound antibodies were revealed using goat anti-rabbit conjugated to ultra-small colloidal gold (US) diluted 1∶25 PBS with 0.1% acetylated BSA (BSA-c™) (Aurion, Wageningen, The Netherlands). After several washes in PBS/0,1%BSA followed by PBS, the antigen-antibody complexes were stabilized on 1% glutaraldehyde in PBS for 5 min After several washes in PBS followed by water colloidal gold silver enhancement was performed using R-Gent™ (Aurion) for 30 min resulting in an average particle size of 10 nm. Thereafter, sections were carefully washed 5 times (5 min each) with water, stained and embedded in a solution containing 4 parts of 2% methyl cellulose (Sigma-Aldrich # M-6385) and one part of 2% uranyl acetate. Sections were observed using a Philips CM 120 transmission electron microscope at an accelerating voltage of 100 kV.

For immunofluorescence the primary antibody was detected with a goat anti-rabbit IgG coupled to AlexaFluor 488 diluted in blocking buffer. Sections were counterstained with DAPI (4′,6-diamidino-2-phenylindole) (1 µg/ml, Sigma) and embedded in Aqua-Poly/Mount (Polysciences # 18606-20) medium. The results were examined using a DM400B Leica epifluorescence microscope connected to a Cool Snap camera (Roper).

#### Quantification of gold particles

The number of particles per µm^2^ was quantified for 15–20 rod nuclei for each immunolabelling experiment. The regions of interest were delimited and their surfaces were measured using the ImageJ software (http://rsbweb.nih.gov/ij/). Gold particles were counted within each region of interest after high pass filtering of the images and by using an automated peak search algorithm (Imaging Science, Berlin, Germany).
